# Epidemiological investigation of adolescent idiopathic scoliosis and evaluation of the therapeutic effect of an integrated sports and medicine rehabilitation strategy for scoliosis treatment

**DOI:** 10.3389/fpubh.2026.1879082

**Published:** 2026-07-06

**Authors:** Xinxuan Xu, Jiyuan Li, Lei Jiang, Yang Qiu, Gang Wu, Jing Jin, Gaonian Zhao

**Affiliations:** 1 Taizhou School of Clinical Medicine, Nanjing Medical University, Taizhou, Jiangsu, China; 2 Department of Rehabilitation Medicine, Nanjing Drum Tower Hospital, Nanjing, Jiangsu, China; 3 Department of Orthopedics, Taizhou People’s Hospital, Taizhou, Jiangsu, China

**Keywords:** adolescent idiopathic scoliosis, Cobb angle, conservative management, epidemiology, integrated sports-medicine rehabilitation, quality of life

## Abstract

**Background:**

Adolescent idiopathic scoliosis (AIS) is a common spinal deformity with significant health impacts. This study aimed to investigate the epidemiology of AIS in a Chinese region and evaluate the efficacy of an integrated sports-medicine rehabilitation (ISMR) strategy.

**Methods:**

This study had two parts. First, a cross-sectional school-based screening investigated AIS epidemiology in adolescents aged 11–19 in Taizhou. Second, a retrospective cohort study evaluated the efficacy of an ISMR strategy. Patients with AIS were allocated to either a Conventional Rehabilitation (CR) group or an ISMR group based on their historical rehabilitation protocol. All received 12 weeks of conservative management. Assessments performed before and after the treatment included Cobb angle, vertebral rotation, apical vertebral translation, trunk rotation angle (ATR), and SRS-22 quality of life scores.

**Results:**

A total of 66,989 individuals were screened. The overall prevalence was higher in females than males (0.35% vs. 0.16%, *p* < 0.001). For the rehabilitation trial, 138 patients were enrolled (CR: *n* = 71; ISMR: *n* = 67). After treatment, the ISMR group showed statistically significant but modest improvements in Cobb angle (13.66 ° vs. 15.04 °, *p* = 0.016), vertebral rotation (1.38 ° vs. 1.59 °, *p* = 0.026), apical translation (20.87 mm vs. 21.89 mm, *p* = 0.033), and ATR (4.84 ° vs. 5.20°, *p* = 0.001) compared to the CR group. The ISMR group also demonstrated statistically and clinically relevant improvements in all SRS-22 domains, including function, pain, self-image, and mental health (all *p* < 0.05).

**Conclusion:**

This study delineates local epidemiological patterns of AIS and demonstrates that a structured, ISMR strategy is more effective than CR in improving spinal alignment and quality of life for conservatively managed patients.

## 1 Introduction

Adolescent idiopathic scoliosis (AIS) is a significant public health issue affecting approximately 2–3% of adolescents globally. It is characterized by an abnormal lateral curvature of the spine, which can lead to physical deformities, impaired respiratory function, and psychosocial problems, including anxiety and low self-esteem (1). Untreated AIS may progress, increasing the risk of surgical treatment and causing long-term complications, with profound psychosocial impacts extending to adolescents’ quality of life and social interactions (2). Therefore, conducting epidemiological surveys on AIS is crucial as it systematically reveals the prevalence, distribution, and characteristics of the disease, providing essential evidence for developing targeted screening, treatment, and management strategies (3, 4).

Currently, various rehabilitation strategies are employed in the management of AIS. Physical therapy aims to improve spinal alignment, flexibility, and muscle strength through targeted exercises, thereby alleviating common functional limitations (5, 6). Rehabilitation training focuses on specific movement patterns to enhance posture control and spinal stability (7, 8). Posture management and adjustment during daily activities help reduce spinal loading and prevent curve progression (9). Psychological rehabilitation is also important, aiming to address body image concerns and anxiety (10). However, these treatments are often implemented in isolation, lacking an integrated framework that can synergistically address the multifaceted needs of AIS patients.

Existing studies exhibit significant deficiencies in integrating traditional medical approaches with comprehensive rehabilitation methods (11). Most protocols focus solely on a single dimension such as exercise, medicine, or psychology, failing to fully leverage the potential advantages of multimodal combined treatments. Sports-medicine integrated rehabilitation, as a multidisciplinary strategy, combines physical therapy, rehabilitation training, posture management, and psychological rehabilitation, aiming to comprehensively improve patient outcomes (12, 13). This strategy seeks to fully meet the physical and mental rehabilitation needs of patients, with the goal of improving treatment results and overall health (14).

This study aims to investigate the epidemiology of AIS in the Chinese population and evaluate the therapeutic efficacy of a new multimodal rehabilitation program. Such an integrated framework may simultaneously advancing more general public health objectives by enhancing rehabilitation results. The findings will provide evidence for this integrated approach, potentially serving as a reference for future treatment paradigms, thereby improving the health and well-being of adolescents affected by AIS.

## 2 Materials and methods

### 2.1 Study design

This study comprised two distinct components. A cross-sectional school-based screening was conducted from September 2019 to June 2022 during the annual student physical examination in the Taizhou area to determine the prevalence and age/sex distribution of AIS. A retrospective cohort study was performed to compare the efficacy of an integrated sports-medicine rehabilitation (ISMR) strategy versus conventional rehabilitation (CR). Patients who were diagnosed with AIS through the screening and met the eligibility criteria were assigned to either the CR group or the ISMR group based on the rehabilitation protocol they had received as part of routine clinical practice (non-randomized, historical allocation). An *a priori* power analysis was conducted for the primary outcome—the absolute change in Cobb angle from baseline to Week 12. Based on previously published data on conservative AIS treatment (15), it was assumed that the CR group would have a mean Cobb angle reduction of 1.0 ° with a standard deviation of 2.8 °, and that a between-group difference of 1.3 ° (i.e., an additional reduction of 1.3 ° in the ISMR group) would represent a clinically meaningful effect (Cohen’s *d* ≈ 0.46). Using a two-sided independent-samples *t*-test with *α* = 0.05 and power of 0.8, a minimum of 64 patients per group was required. As this was a retrospective study, we screened all consecutive AIS cases recorded between June 2019 and June 2022 that met the eligibility criteria and had complete baseline and 12-week follow-up data. A total of 138 patients were ultimately included (CR group: *n* = 71; ISMR group: *n* = 67), exceeding the minimum sample size. Since all patients had already completed treatment, no dropouts occurred. The study protocol was approved by the Ethics Committee of Taizhou People’s Hospital (approval no. KY 2022-137-01), and written informed consent was obtained from the families of all minor subjects.

### 2.2 Investigation methods and diagnostic criteria

The screening was integrated into the government-mandated annual physical examination program covering all regular secondary schools in Taizhou. Therefore, the target population comprised all registered adolescents aged 11–19 years in the region.

A standardized three-step screening method was adopted for the epidemiological investigation and case confirmation of AIS, including visual inspection, the Adam’s forward bending test combined with angle measurement, and full spine X-ray examination.

The first step was visual inspection. Subjects stood naturally with feet shoulder-width apart, arms hanging loosely at their sides, palms facing inward, and their backs exposed to the examiner standing behind them. The examiner observed shoulder levelness, symmetry of the bilateral scapular lower angles and lumbar dimples, iliac crest height, and the line of the spinous processes. Any obvious asymmetry was considered a suspected positive result, and the subject proceeded to the second step.

The second step was the Adam’s forward bending test. With the subject’s back exposed and facing the light source, knees extended, and the trunk slowly bent forward to 90 °, the examiner observed the spine from behind along its midline. If an asymmetrical hump (rib or lumbar hump) was detected, a scoliometer was used to measure the angle of trunk rotation (ATR). The maximum deviation angle and its location were recorded. Subjects with an ATR greater than 4 ° proceeded to the third step.

The third step was radiographic confirmation. All participants with ATR ≥ 4 ° underwent standing full-spine anteroposterior and lateral X-rays, taken by radiographic technicians from our hospital. Two senior orthopedic surgeons independently reviewed the radiographs and measured the Cobb angle using the Cobb method. A diagnosis of AIS was made when the Cobb angle was ≥10 °. Patients with radiographic or clinical evidence of non-idiopathic scoliosis (e.g., congenital vertebral anomalies, neuromuscular disorders, trauma) were excluded. If the measurement discrepancy between the two surgeons exceeded 5 °, the final value was determined by consensus. The intraclass correlation coefficient (ICC) for Cobb angle measurements between the two surgeons was 0.93 (95% CI: 0.89–0.97), indicating excellent interobserver reliability.

### 2.3 Inclusion and exclusion criteria

#### 2.3.1 Epidemiological investigation part

*Inclusion criteria*: Adolescents aged 11 to 19 years who participated in the annual student physical examination in the Taizhou area.

#### 2.3.2 Rehabilitation efficacy study part

This study aims to evaluate the efficacy of an integrated sports-medicine rehabilitation strategy.

*Inclusion criteria*: Patients initially identified through the aforementioned epidemiological screening and clinically diagnosed with AIS strictly according to the Scoliosis Research Society (SRS) criteria (16, 17); no prior history of any specific treatment for scoliosis; family members signed informed consent, committing to actively cooperate with a 12-week rehabilitation program.

*Exclusion criteria*: Non-idiopathic scoliosis secondary to neuromuscular diseases, congenital vertebral deformities, trauma, or tumors; prior history of spine or lower limb surgery; presence of severe cognitive or communication impairments that prevent understanding or cooperation with rehabilitation training; coexisting other serious chronic diseases expected to interfere with the rehabilitation process or quality of life assessment.

### 2.4 Assessment of treatment effects

Before treatment and after 12 weeks of treatment, a rehabilitation physician who is unaware of the patients’ group assignments conducts blinded assessments for both groups. The assessment covers three aspects: imaging, physical examination, and patient-reported outcomes.

Imaging assessment is based on standard standing full-spine anteroposterior and lateral X-rays taken before and after treatment. Cobb angle serves as the primary metric, defined as the angle between the perpendicular lines from the upper endplate of the upper-end vertebra and the lower endplate of the lower-end vertebra in the scoliosis curve. Vertebral rotation angles are assessed using the widely used Nash-Moe semi-quantitative method, which classifies the degree of rotation from grade 0 to IV based on the displacement of the pedicles relative to the vertebral side walls on the anteroposterior X-ray images. Apical Vertebral Translation (AVT) is measured as the horizontal distance from the midpoint of the apical vertebra in the scoliosis curve to the Central Sacral Vertical Line (CSVL), recorded in millimeters.

Physical examination involves using a unified scoliosis measurement instrument (Scoliometer, Baseline®, Fabrication Enterprises, China) to measure the trunk rotation angle during Adam’s forward bending position. Measurements are taken at the thoracic kyphosis peak and lumbar lordosis peak, with three consecutive measurements averaged.

Patient-reported outcomes are evaluated using the Scoliosis Research Society-22 questionnaire (SRS-22) (18). This questionnaire covers five dimensions: function/activity (5 items), pain (5 items), self-image (5 items), mental health (5 items), and satisfaction with treatment (2 items), totaling 22 items. Each item uses a Likert 5-point scale (1 represents “worst/never,” 5 represents “best/always”). Scores for each dimension are calculated as the mean score of its items, with a total score range of 22 to 110 points. Higher scores indicate better quality of life.

### 2.5 Rehabilitation methods

All patients in this study received non-surgical conservative rehabilitation treatment. According to the established protocol, patients were divided into two groups and received standardized rehabilitation treatments for 12 weeks, three times per week, each session lasting approximately 60 min. The selection and dosing of rehabilitation modalities were based on current evidence-based guidelines for adolescent idiopathic scoliosis (AIS) management, particularly emphasizing multimodal approaches that integrate physical, educational, and psychological components (19). During the 12-week treatment period, no patient in either group wore a brace or received any other form of orthotic management for scoliosis.

The CR Group’s treatment plan focused on basic exercise therapy and health education. The content included diaphragmatic breathing training guided by therapists to optimize breathing patterns. Standardized resistance and stability exercises targeting the core muscles of the waist and abdomen as well as lower limb strength, with each set of exercises repeated 15–20 times, completing 2-3 sets. Posture protection education was provided, guiding patients to maintain a neutral spine position during daily sitting, standing, and walking, and avoiding exaggerated movements involving abrupt twisting or lateral flexion of the trunk. Basic psychological support was provided, explaining to patients the normal muscle soreness and fatigue that may occur during rehabilitation to alleviate anxiety.

The ISMR Group implemented a structured multimodal integrated program on top of the conventional regimen. This program consisted of five synergistic modules: individualized physical therapy, where specific three-dimensional self-correction exercises and spinal flexibility practices were taught by physical therapists based on the patient’s Cobb angle and apical vertebra position, with patients required to complete a 20-min consolidation training at home daily; specialized rehabilitation training, supervised progressive spinal derotation exercises and neuromuscular control training using equipment such as resistance bands and balance pads, with intensity controlled at a rating of perceived exertion score of 13–15 out of 20; systematic posture management and behavioral reshaping, including daily posture guidance and optimization suggestions for learning environments and backpack weights; structured health education classes held once a week, covering knowledge about scoliosis, self-management, and pain coping strategies; group psychological rehabilitation sessions held every 2 weeks, led by a psychological counselor to promote experience sharing and social support among patients, actively managing body image-related issues. Both groups’ treatments were carried out by a fixed rehabilitation team in the same environment to ensure consistency of the treatment.

### 2.6 Statistical analysis

Data analysis was performed using SPSS 29.0 statistical software (SPSS Inc., Chicago, IL, United States). Categorical data were presented as [*n* (%)] forms. For cases where the theoretical frequency *T* ≥ 5, the Chi-square test was used. For samples with 1 ≤ *T* < 5, Fisher’s exact test was applied. Continuous variables were tested for normality using the Shapiro–Wilk method. Normally distributed data were presented as mean ± standard deviation (*X* ± *s*) and compared using the independent-samples *t*-test. Non-normally distributed data were presented as median (25th, 75th percentiles) and compared using the Mann–Whitney *U* test. A two-tailed *p* < 0.05 was considered statistically significant. The primary outcome (change in Cobb angle) and all continuous secondary outcomes were analyzed using analysis of covariance (ANCOVA). All analyses of secondary outcomes are considered exploratory; therefore, no correction for multiplicity was applied.

## 3 Result

### 3.1 Epidemiological characteristics of AIS

The distribution of AIS by age shows that across nine age groups ranging from 11 to 19 years, a total of 66,989 individuals were tested (Table 1). The positivity rate varied significantly across these age groups, starting from 0.11% at age 11, reaching its lowest at 0.10% for 12-year-olds, and then fluctuating slightly until it peaked at 0.92% for 18-year-olds before dropping to 0.44% for 19-year-olds. Notably, the positivity rate showed an increase from age 15 onwards, with rates being particularly high for ages 17 and 18.

**Table 1:** Distribution of adolescent idiopathic scoliosis by age.

Age (years)	11	12	13	14	15	16	17	18	19
Individuals tested	13,489	11,698	9,824	8,723	7,615	5,213	4,783	3,148	2,496
Positive cases	15	12	12	11	21	16	31	29	11
Male	8	6	4	5	8	4	13	11	4
Female	7	6	8	6	13	12	18	18	7
Positivity rate (%)	0.11	0.10	0.12	0.13	0.28	0.31	0.65	0.92	0.44

In the comparison of the prevalence of scoliosis between different genders, a total of 39,548 males and 27,441 females were tested, resulting in 63 positive cases among males and 95 among females, with prevalence rates of 0.16 and 0.35%, respectively, (Table 2). The result indicated a significant difference between the two groups (*p* < 0.001), demonstrating that the prevalence rate of scoliosis was significantly higher in females compared to males. This suggests that female individuals may have a greater susceptibility to scoliosis, highlighting the importance of considering gender differences in the screening and management strategies for this condition.

**Table 2:** Comparison of the prevalence of scoliosis between different genders.

Gender	Individuals tested	Positive cases	Prevalence (%)	*χ* ^2^	*p*
Male	39,548	63	0.16	24.049	<0.001
Female	27,441	95	0.35		

The distribution of Cobb angles in AIS patients across different ages shows a varied pattern with respect to the severity of spinal curvature (Table 3). For patients aged 11, there were 10 cases with Cobb angles less than 35 degrees and 5 cases with angles greater than or equal to 35 degrees out of a total of 15 cases. As age progressed, the number of cases with Cobb angles less than 35 degrees generally increased, peaking at age 17 and 18 with 25 and 26 cases respectively, while the number of severe cases (Cobb angle ≥35 °) remained relatively low, except for age 17 where it reached 6 cases. Notably, among the oldest group (age 19), there were fewer total cases but a relatively high proportion had Cobb angles greater than or equal to 35 degrees. These observations suggest that while the majority of cases tend to have less severe spinal curvatures (Cobb angle <35 °), there is still a significant presence of more severe cases especially around mid to late adolescence, indicating the importance of early detection and intervention to manage the progression of scoliosis.

**Table 3:** Distribution of cobb angles in AIS patients of different ages.

Age/Angle	<35 °	≥35 °	Total
11	10	5	15
12	10	2	12
13	9	3	12
14	6	5	11
15	17	4	21
16	12	4	16
17	25	6	31
18	26	3	29
19	7	4	11

### 3.2 Comparison of rehabilitation outcomes between CR and ISMR groups

In the general information comparison of patients with idiopathic scoliosis between the CR group and the ISMR group, no significant differences were observed in age distribution, gender, menarchal status, Cobb angle classification, BMI-*Z* classification, curve type, curve location, or Risser sign (all *p* > 0.05; Table 4). These results indicate that the two groups were well-matched at baseline, providing a solid foundation for further investigation of treatment effects.

**Table 4:** General information of patients with idiopathic scoliosis.

Index	CR group (*n* = 71)	ISMR group (*n* = 67)	*t*/*χ*^2^	*p*
Age (years)			0.011	0.918
<15 years	28 (39.44%)	27 (40.30%)		
≥15 years	43 (60.56%)	40 (59.70%)		
Gender			0.084	0.772
Male	29 (40.85%)	29 (43.28%)		
Female	42 (59.15%)	38 (56.72%)		
Menarchal status in females (premenarchal/postmenarchal)	8 (19.05%)/34 (80.95%)	7 (18.42%)/31 (81.58%)	0.005	0.943
Cobb angle			0.114	0.736
<35°	49 (69.01%)	48 (71.64%)		
≥35°	22 (30.99%)	19 (28.36%)		
BMI-*Z* score			0.190	0.909
Underweight	6 (8.45%)	7 (10.45%)		
Normal range	57 (80.28%)	52 (77.61%)		
Overweight	8 (11.27%)	8 (11.94%)		
Curve type			0.949	0.814
Thoracic	32 (45.07%)	27 (40.30%)		
Thoracolumbar	18 (25.35%)	22 (32.84%)		
Lumbar	12 (16.90%)	10 (14.93%)		
Double	9 (12.68%)	8 (11.94%)		
Curve location			0.369	0.832
Thoracic T5–T11	33 (46.48%)	29 (43.28%)		
Thoracolumbar T12–L1	23 (32.39%)	25 (37.31%)		
Lumbar L2–L4	15 (21.13%)	13 (19.40%)		
Risser sign (0–2/3–5)	46 (64.79%)/25 (35.21%)	42 (62.69%)/25 (37.31%)	0.066	0.797

In the comparison of spinal parameters before treatment, there were no significant differences between the CR and ISMR groups in Cobb angle (*p* = 0.897), vertebral rotation (*p* = 0.348), apical vertebral translation (*p* = 0.501), or trunk rotation angle (*p* = 0.288; Table 5), confirming baseline comparability. After 12 weeks of treatment, ANCOVA adjusting for baseline values demonstrated that the ISMR group had significantly lower adjusted Cobb angle (adjusted mean difference −1.34 °, 95% CI −2.21 ° to −0.47 °, *p* = 0.016, Supplementary Table 1), apical vertebral translation (adjusted difference −0.99 mm, *p* = 0.033), and trunk rotation angle (adjusted difference −0.38 °, *p* = 0.001) compared with the CR group. Sensitivity analyses using changes from baseline for these continuous outcomes yielded consistent results (all *p* < 0.05). After treatment, the ISMR group showed significantly lower Nash-Moe grades compared with the CR group (median 1.00 [IQR 1.00–2.00] vs. 2.00 [1.00–2.00], *U* = 1957.0, *p* = 0.048). Overall, these findings indicate that the ISMR strategy was associated with greater improvements in spinal alignment relative to CR after accounting for baseline values.

**Table 5:** Comparison of spinal parameters before and after treatment.

Index	CR group (*n* = 71)	ISMR group (*n* = 67)	*t*/*U*	*p*
Before treatment				
Cobb angle (°)	16.58 ± 3.41	16.49 ± 4.27	0.130	0.897
Vertebral rotation angle (Nash-Moe grade)	2.00 [2.00, 2.00]	2.00 [1.00, 2.00]	2182.000	0.348
Apical vertebral translation (mm)	23.55 ± 3.47	23.17 ± 2.98	0.675	0.501
Trunk rotation angle (°)	7.66 ± 0.19	7.69 ± 0.15	1.066	0.288
After treatment				
Cobb angle (°)	15.04 ± 2.62	13.66 ± 3.84	2.454	0.016
Vertebral rotation angle (Nash-Moe grade)	2.00 [1.00, 2.00]	1.00 [1.00, 2.00]	1957.000	0.048
Apical vertebral translation (mm)	21.89 ± 2.95	20.87 ± 2.56	2.157	0.033
Trunk rotation angle (°)	5.20 ± 0.12	4.84 ± 0.87	3.376	0.001

In the comparison of quality of life scores before treatment between the two groups, no significant differences were observed in functional activity (*p* = 0.648), pain (*p* = 0.723), self-image (*p* = 0.368), or mental health (*p* = 0.818; Table 6), confirming baseline comparability. After 12 weeks, ANCOVA adjusting for baseline scores demonstrated that the ISMR group had significantly higher adjusted scores in functional activity (adjusted mean difference 0.20, 95% CI 0.04–0.36, *p* = 0.013), pain (adjusted difference 0.26, 95% CI 0.06–0.46, *p* = 0.011), self-image (adjusted difference 0.22, 95% CI 0.08–0.36, *p* = 0.003), and mental health (adjusted difference 0.26, 95% CI 0.04–0.48, *p* = 0.022) compared with the CR group (Supplementary Table 2). Treatment satisfaction, assessed only after treatment, was also significantly higher in the ISMR group (mean difference 0.27, 95% CI 0.07–0.47, *p* = 0.007, independent-samples *t*-test). Sensitivity analyses using changes from baseline yielded consistent results for all repeated domains (all *p* < 0.05). These findings suggest that the ISMR strategy was associated with greater improvements in health-related quality of life and treatment satisfaction compared with CR after accounting for baseline scores.

**Table 6:** Quality of life score.

Index	CR group (*n* = 71)	ISMR group (*n* = 67)	*t*	*p*
Before treatment				
Functional activity	4.21 ± 0.42	4.18 ± 0.53	0.458	0.648
Pain	4.52 ± 0.48	4.49 ± 0.52	0.355	0.723
Self-image	3.02 ± 0.51	3.11 ± 0.69	0.903	0.368
Mental health	3.99 ± 0.21	4.01 ± 0.64	0.231	0.818
After treatment				
Functional activity	4.41 ± 0.37	4.59 ± 0.47	2.530	0.013
Pain	4.57 ± 0.65	4.81 ± 0.47	2.565	0.011
Self-image	3.59 ± 0.25	3.79 ± 0.47	3.045	0.003
Mental health	4.35 ± 0.65	4.59 ± 0.58	2.315	0.022
Treatment satisfaction	3.81 ± 0.51	4.08 ± 0.65	2.721	0.007

## 4 Discussion

This study provides new epidemiological insights into AIS among the population in China, and more importantly, focuses on the evaluation of structured, multimodal conservative rehabilitation strategies. Our findings contribute to understanding the distribution patterns of AIS and support the clinical value of integrating principles of sports and medicine into a comprehensive non-surgical management framework to improve biomechanical alignment and patient-centered outcomes.

Our epidemiological survey confirmed the dominance of females in the prevalence of AIS, a phenomenon consistently observed across different populations. The underlying etiology of this gender disparity remains multifactorial and not fully understood, but it is often attributed to complex interactions involving genetic predisposition, different growth patterns and rates, and the influence of pubertal hormones (20, 21). Studies suggest that rapid growth in females, combined with potential hormone-mediated effects on ligament laxity and muscle development, may lead to a higher susceptibility to spinal deformities (22). While our overall rates confirm this trend, a detailed analysis targeting different age groups showed that this difference is not consistent across all adolescent years but is more pronounced during a specific period in mid-adolescence. This nuance aligns with the concept that risk is dynamic and may peak during the phase of fastest bone growth, emphasizing the importance of timing in screening programs (23). The observed distribution of curve severity, with a larger proportion of Cobb angles in older adolescents, further underscores the progression potential of AIS in untreated or poorly managed cases. This progression highlights the critical public health window for early detection and effective treatment to reduce the risk of deformities progressing (24).

The core finding of this study is that in a conservative management setting, the ISMR program demonstrates efficacy compared to CR. The 1.4 ° Cobb angle difference is small and within measurement error, warranting cautious interpretation. However, consistent improvements across multiple secondary outcomes support ISMR’s potential relevance. Future studies with longer follow-up are needed to confirm clinical meaningfulness. Even a slight improvement in the Cobb angle may delay progression, and manifest as a meaningful enhancement in the patient-reported dimensions of quality of life. In the ISMR group, improvements in key radiological and physical examination parameters indicate meaningful structural and functional benefits. This contradicts any simplistic notion that conservative treatment merely prevents progression (25, 26). Instead, it showcases the potential for active correction, particularly when employing a multifaceted approach. The mechanisms behind these improvements may stem from the synergistic effects of the ISMR components. Prescribed three-dimensional self-correction exercises target specific spinal segments to promote derotation and realignment, while neuromuscular control and stability training enhance the trunk’s “muscular corset” ability to maintain correct posture against gravity and habitual forces (27). This aligns with the principles of Physiotherapeutic Scoliosis-Specific Exercises (PSSE), which have been evidenced to reduce curve progression and improve postural alignment (28). Our ISMR protocol goes beyond PSSE by systematically integrating these exercises with behavioral posture re-education and psychological support, creating a more comprehensive and potentially more sustainable treatment. Previous studies on exercise-based treatments have reported varied outcomes, often limited by the heterogeneity in exercise protocols, intensity, and adherence (29, 30). The structured, supervised, and multimodal nature of our ISMR program may be why its effects are more robust, ensuring proper technique and comprehensive engagement with factors affecting scoliosis.

In ISMR participants, improvements were also significantly better across all domains of health-related quality of life, which is equally important. The SRS-22 questionnaire documented patients’ lived experiences, including function, pain, self-image, and mental health. The greater gains in the ISMR group indicate that the benefits of this integrated strategy extend beyond radiographic metrics, directly translating into enhanced daily well-being. Functional improvements may be attributed to increased spinal mobility, strength, and movement confidence gained through tailored exercises (31). Pain relief could be associated with better spinal biomechanics, reduced muscle imbalances, and pain coping strategies provided in the educational modules (32). Differences observed in self-image and mental health can be directly attributed to specialized psychological rehabilitation sessions, which provide a safe space for addressing body image issues, reducing condition-related anxiety, and fostering peer support (33). Isolated exercise programs, while beneficial for physical health, often neglect this crucial psychosocial dimension. Our findings align with a growing body of literature that emphasizes the necessity of addressing the psychological burden of AIS (34). They strongly suggest that effective rehabilitation must be biological, psychological, and social, actively addressing the emotional and self-perception challenges inherent in visible deformities during adolescence. The correlation between the ISMR strategy and better post-treatment HRQoL scores further reinforces the notion that the comprehensiveness and patient engagement of an treatment are intrinsically linked to superior overall outcomes.

Despite these encouraging results, several limitations of this study must be acknowledged. First, this is a single-center study conducted in a specific region of China, which may limit the generalizability of the epidemiological findings and rehabilitation outcomes to other populations with different genetic backgrounds, healthcare systems, or cultural attitudes toward rehabilitation. Second, although the sample size was pre-calculated and met the minimum requirements, a larger multicenter trial would provide more reliable and definitive evidence. Third, while the 12-week treatment period was sufficient to demonstrate significant changes, it is relatively short. The long-term sustainability of these improvements, their impact on final skeletal maturity outcomes, and the potential to reduce lifetime progression risk remain unknown and require investigation through extended follow-up. Fourth, the conventional rehabilitation provided to the control group, although standard in our environment, may differ from those in other regions or studies, which could influence the magnitude of the observed inter-group differences. Fifth, some of the assessment methods used in this study, although they include X-ray examination as an objective standard, still contain certain subjective components. The non-randomized design and retrospective allocation may have introduced selection bias, although baseline characteristics were comparable between groups. Finally, the intensive, therapist-supervised nature of the ISMR program, while being an advantage for enhancing efficacy, poses challenges for resource allocation and widespread implementation. Future research should aim to replicate these findings in different settings, investigate the optimal duration and necessary intensity of such programs, evaluate long-term outcomes, and explore ways to enhance accessibility and cost-effectiveness without compromising core efficacy. The ISMR protocol inherently involved more frequent supervision, additional home exercises, and structured psychological sessions compared with CR, creating differences in total therapeutic exposure and patient attention. This risk of performance bias, inherent to the non-randomized design, is acknowledged as a limitation of this study.

## 5 Conclusion

This epidemiological investigation delineates age- and gender-specific patterns of adolescent idiopathic scoliosis in a regional Chinese cohort, providing data to inform targeted screening. Furthermore, the study suggests that a structured, multimodal sports-medicine integrated rehabilitation strategy may offer additional short-term benefits in spinal alignment and health-related quality of life compared with conventional rehabilitation, although the modest radiographic improvements should be interpreted with caution and their clinical significance requires further validation.

## Data Availability

The raw data supporting the conclusions of this article will be made available by the authors, without undue reservation.
