# Supplementary material for: Epidemiological investigation of adolescent idiopathic scoliosis and evaluation of the therapeutic effect of an integrated sports and medicine rehabilitation strategy for scoliosis treatment
Source: Front Public Health. 2026 Jul 6;14:1879082. doi: 10.3389/fpubh.2026.1879082 (PMC13381731; doi:10.3389/fpubh.2026.1879082)
Supplement: Supplementary file 1 [file Table_1.docx]

Supplementary Table 1 Comparison of spinal parameters after 12-week treatment adjusted for baseline values

| Index | CR Group (n=71) Adjusted Mean (SE) | ISMR Group (n=67) Adjusted Mean (SE) | Adjusted Mean Difference (95% CI) | F | P |
| --- | --- | --- | --- | --- | --- |
| Cobb angle (°) | 15.02 (0.31) | 13.68 (0.32) | −1.34 (−2.21, −0.47) | 6.02 | 0.016 |
| Apical vertebral translation (mm) | 21.87 (0.34) | 20.88 (0.35) | −0.99 (−1.90, −0.08) | 4.62 | 0.033 |
| Trunk rotation angle (°) | 5.21 (0.07) | 4.83 (0.07) | −0.38 (−0.56, −0.20) | 11.40 | 0.001 |
