# Supplementary material for: Epidemiological investigation of adolescent idiopathic scoliosis and evaluation of the therapeutic effect of an integrated sports and medicine rehabilitation strategy for scoliosis treatment
Source: Front Public Health. 2026 Jul 6;14:1879082. doi: 10.3389/fpubh.2026.1879082 (PMC13381731; doi:10.3389/fpubh.2026.1879082)
Supplement: Supplementary file 2 [file Table_2.docx]

Supplementary Table 2 Comparison of SRS-22 scores after 12-week treatment adjusted for baseline values

| Index | CR Group (n=71) Adjusted Mean (SE) | ISMR Group (n=67) Adjusted Mean (SE) | Adjusted Mean Difference (95% CI) | P |
| --- | --- | --- | --- | --- |
| Function | 4.40 (0.05) | 4.60 (0.05) | 0.20 (0.04, 0.36) | 0.013 |
| Pain | 4.56 (0.07) | 4.82 (0.07) | 0.26 (0.06, 0.46) | 0.011 |
| Self-image | 3.58 (0.04) | 3.80 (0.05) | 0.22 (0.08, 0.36) | 0.003 |
| Mental health | 4.34 (0.07) | 4.60 (0.07) | 0.26 (0.04, 0.48) | 0.022 |
| Treatment satisfaction^1^ | 3.81 (0.06) | 4.08 (0.08) | 0.27 (0.07, 0.47) | 0.007 |

^1^Treatment satisfaction was analyzed by independent-samples t‑test (no baseline value). All other domains were analyzed by ANCOVA with baseline scores as covariates.
